# Adult-diagnosed and childhood-diagnosed attention deficit/hyperactivity disorder: cognitive and environmental contributions to symptom severity across different age of diagnosis

**DOI:** 10.3389/fpsyt.2026.1782999

**Published:** 2026-04-30

**Authors:** Simin Kang, Zhao Fu, Qiurong Li, Li Yang, Qingjiu Cao

**Affiliations:** 1 Peking University Sixth Hospital, Peking University Institute of Mental Health, NHC Key Laboratory of Mental Health (Peking University), National Clinical Research Center for Mental Disorders (Peking University Sixth Hospital), Beijing, China; 2 Beijing Key Laboratory for Big Data Innovative Application of Child and Adolescent Mental Disorders, Beijing, China; 3 NO 984 Hospital of PLA, Beijing, China

**Keywords:** adult ADHD, adult-diagnosed, environmental factors, executive function, symptom severity

## Abstract

**Background:**

Attention deficit/hyperactivity disorder (ADHD) is characterized as a neurodevelopmental disorder onset in childhood. However, accumulating evidence suggests that some adults may fulfill current diagnostic criteria for ADHD despite reporting no clinically significant symptoms during childhood. It remains unclear whether executive function and environmental factors relate differently to symptom severity across different age of diagnosis.

**Methods:**

Seventy-two adults with ADHD were recruited and classified into childhood-diagnosed (N = 37) and adult-diagnosed (N = 35) groups using structured diagnostic interviews and retrospective symptom ratings. ADHD symptoms were assessed using the Adult ADHD self-report scale, executive function with the Behavior Rating Inventory of Executive Function-Adult Form, emotional symptoms via the Self-Rating Anxiety Scale and Self-Rating Depression Scale, and environmental factors using the Childhood Trauma Questionnaire, Connor-Davidson Resilience Scale, and Parental Bonding Instrument. Hierarchical multiple regression and moderation analyses were conducted to evaluate independent contributions and diagnosis-specific effects on ADHD core symptoms.

**Results:**

There was no significant age difference between the adult-diagnosed and childhood-diagnosed ADHD groups (25.63 ± 4.89 vs. 26.32 ± 4.69 years). Compared to the childhood-diagnosed group, adults with adult-diagnosed ADHD showed lower childhood symptoms but similar adulthood severity, alongside superior self-monitoring and significantly more psychiatric comorbidities and emotional distress. Executive function was the strongest and most consistent predictor of both inattention (*β* = 0.64, *p* < 0.001) and hyperactivity-impulsivity symptoms (*β* = 0.47, *p* < 0.001), whereas environmental factors contributed minimal additional explanatory power, with a modest association between parental overprotection and inattention (*β* = 0.28, *p* = 0.016). No diagnosis-specific moderation effects were observed (all *p* > 0.05).

**Conclusions:**

These findings suggest that while adult-diagnosed ADHD presents with a complex clinical profile, executive function and environmental factors do not play a primary role specific to the age of diagnosis.

## 1 Introduction

Attention-deficit hyperactivity disorder (ADHD) is a neurodevelopmental disorder, with symptoms present before the age of 12 (1). However, recent studies have identified some adults who meet current criteria for ADHD despite having no history of childhood ADHD, a condition termed “adult-onset ADHD” or “late-onset ADHD” (2–4). A recent systematic review on the worldwide prevalence of ADHD in adults found that the prevalence of persistent adult ADHD (i.e. those diagnosed with ADHD in childhood and still experiencing significant symptoms into adulthood) remained stable at 2.58%, consistent with prior studies (5). When the childhood-onset criterion was excluded, however, the estimated prevalence more than doubled to 6.76%. This means that most adults who currently exhibit ADHD symptoms did not show these symptoms in childhood, which challenges the specificity of ADHD symptoms.

One of the most fundamental questions raised by these findings is whether adult-diagnosed ADHD represents the same disease continuum as childhood-diagnosed ADHD or reflects distinct pathogenic mechanisms or heterogeneous subtypes (3, 6). In this context, environmental factors and/or higher cognitive ability have often been proposed as potential explanatory frameworks for late-diagnosed ADHD (7–9). Indeed, in certain cases, lower environmental demands in childhood, along with greater individual cognitive resources or supportive family environments, may delay the emergence of clinically impairing symptoms during early development. Such symptoms may become apparent in adolescence and early adulthood, when individuals encounter increased demands for autonomy or higher expectations (10).

Previous studies have extensively investigated the link between environmental factors and ADHD symptom severity, including parenting bonding, adverse childhood experiences, and individuals’ psychological adaptation to stress (11–15). Research has begun to examine whether these environmental factors are specifically linked to later-emerging ADHD symptoms. Sibley reported that adolescents with late-diagnosed ADHD experienced higher demands from parents and pronounced history of multiple trauma exposure (16). In contrast, a population-based longitudinal study found that adult-diagnosed ADHD had more childhood resources than those with childhood-diagnosed ADHD (8). More recent research found no difference in parenting attitudes between adult-diagnosed and childhood-diagnosed ADHD, yet fear of abandonment was significantly higher in the childhood-diagnosed group (17). Collectively, although environmental factors have been implicated in ADHD symptomatology in adolescence and adulthood, it remains unclear whether their influence is specific to the age of diagnosed (4).

Meta‐analyses have consistently shown that adults with ADHD experience impairments in executive function (10, 18), and these executive dysfunctions are linked to the core symptom domains of inattention and hyperactivity/impulsivity. However, few studies have explicitly examined whether the association between executive function and ADHD symptom expression differs between adult-diagnosed and childhood-diagnosed ADHD, or whether the relationships vary as a function of age of diagnosis. Adults with ADHD frequently present with co-occurring psychiatric disorders, notably major depressive disorder and anxiety disorders, which may increase clinical complexity and potentially intensify the severity of ADHD symptoms (19). Consequently, anxiety and depressive symptoms are important factors to consider in examining the cognitive and environmental correlates of ADHD, especially in adults with heterogeneous ages of diagnosis.

In this study, we examined how environmental factors and executive function relate to ADHD core symptoms in adults with differing histories of clinical diagnosis. We further investigated whether the strength of these associations varies between adult-diagnosed and childhood-diagnosed ADHD.

## 2 Methods and materials

### 2.1 Participants

The study was conducted in Peking University Sixth Hospital between January 2023 and August 2024. Seventy-two participants were included. Inclusion criteria were an age range of 18 to 45 years and a diagnosis of ADHD according to DSM-5. Exclusion criteria included: 1) current diagnosis of other mental disorder; 2) severe physical illness or neurological diseases. The diagnosis of ADHD was confirmed by structured interview according to the Conner’s Adult ADHD Diagnostic Interview (CAADID) based on DSM criteria. Potential comorbidities were screened out using the Mini International Neuropsychiatric Interview (MINI) based on DSM-IV. The study was approved by the Research Ethics Committee of Peking University Sixth Hospital, and all participants provided written informed consent before enrollment.

Childhood-diagnosed ADHD (n = 37) was defined by the fulfillment of all DSM-5 diagnostic criteria for ADHD during both childhood (before 12 years of age) and adulthood (after 18 years of age). Adult-diagnosed ADHD (N = 35) was defined as meeting DSM-5 criteria for ADHD in adulthood, while not meeting criteria for any ADHD subtype in childhood (endorsement of ≤3 symptoms in either domain on the parent-reported ADHD rating scale).

### 2.2 Measures

For adults with ADHD, ADHD core symptoms in adulthood were measured by the Adult ADHD self-report scale (ASRS) consists of two subscales: inattention (0–36 points) and hyperactivity/impulsivity (0–36 points), with a range for the ASRS total score of 0–72 points (20). ADHD symptoms in childhood and adolescence were evaluated using ADHD rating scale (ADHD-RS) by their parents (21). ADHD-RS item ratings were recoded to correspond with symptom counting in clinical practice for participant classification: scores of 0 or 1 were recoded as 0 (absence of the symptom), whereas scores of 2 or 3 were recoded as 1 (presence of the symptom). Sum scores of the recoded ADHD-RS, ranging from 0 to 9 separately for the two symptom domains indicated number of endorsed ADHD symptoms per individual.

Anxiety/depression symptoms were assessed by the Self-Rating Anxiety Scale (SAS) (22) and Self-Rating Depression Scale (SDS) (23), each comprising 20 items. Standard cutoff scores of 50 for the SAS and 53 for the SDS were applied, with higher scores indicating more severe symptom severity.

Executive function was evaluated using the Behavior Rating Inventory of Executive Function-Adult Form (BRIEF-A) (24). The 75 items are divided into nine theoretically and empirically derived scales and two broader indices, which together make up the global executive composite (GEC) score. *T*-scores ≥65 are considered clinically significant and suggest notable executive function difficulties.

Childhood trauma was assessed by the Childhood Trauma Questionnaire (CTQ), a self-report instrument developed to assess experiences of childhood maltreatment across five domains: emotional abuse, physical abuse, sexual abuse, emotional neglect, and physical neglect (25).

Resilience was assessed by using the Connor-Davidson Resilience Scale (CD-RISC), a unidimensional measure that evaluates the capacity to cope with challenges such as change, health issues, and emotional distress (26). The scale comprises 25 items rated on a 5-point Likert scale, with higher scores indicating greater resilience.

Parental bonding was assessed by the Parental Bonding Instrument (PBI), a self-report questionnaire designed to assess individuals’ perceptions of their parents’ behaviors during the first 16 years of life (27). PBI consists of two factors: the care factor and the overprotection factor, and the parental care scores and overprotection scores were calculated.

### 2.3 Statistical analysis

Statistical analyses were conducted using R software (version 4.2.0). Categorical variables were reported as frequencies and percentages, with differences among groups assessed using the chi-square test. Continuous variables were presented as mean and standard deviation, and differences among groups (including symptom severity and psychometric parameters) were analyzed using the independent samples t-test for normally distributed data or Mann-Whitney U-tests for non-normal distributions data. Pearson correlations analysis assessed the bivariate associations between ADHD core symptoms, executive function (GEC), emotional symptoms, and environmental factors (childhood trauma, resilience, parental care and overprotection).

Hierarchical multiple regression models were employed to examine the independent and cumulative effects of executive functioning and environmental variables on ADHD core symptoms. Separate analyses were performed with inattention and hyperactivity-impulsivity symptoms modeled as distinct dependent variables. In Step 1, demographic variables (age, gender, medication status, comorbidity and age of diagnosis) were entered as control variables, with age of diagnosis was coded as 0 = adult-diagnosed ADHD and 1 = childhood-diagnosed ADHD. In Step 2, executive function was added. In Step 3, emotional symptoms were included. In Step 4, environmental factors were added, comprising childhood trauma, resilience, parental care, and parental overprotection. Continuous predictors were mean-centered as a preprocessing step before analysis. To examine whether associations differed by age of diagnosis, interaction terms between the age of diagnosis and each focal variable were tested in separate models. A two-tailed p-value ≤0.05 was considered statistically significant. Given the sample size of N = 72, a sensitivity power analysis was conducted using G*Power 3.1 to determine the minimum detectable effect size for our hierarchical regression models. With alpha = 0.05 and a power of 0.80, the study was equipped to detect a medium effect size of Cohen’s *f*^2^ = 0.113. This indicates that while the study is robust for identifying moderate-to-large effects, it may have limited sensitivity to detect small-to-medium effect sizes.

## 3 Result

### 3.1 Demographic and clinical characteristics

A total of 72 adults, consisting of 35 patients with adult-diagnosed ADHD and 37 patients with childhood-diagnosed ADHD, were recruited. **Table 1** shows demographics, clinical symptoms and psychometric features of the two diagnosis groups. No significant differences were found between the adult-diagnosed and childhood-diagnosed ADHD groups in age (*p* = 0.540), gender (*p* = 0.059), educational years (*p* = 0.858), educational level (*p* = 0.402), employment status (*p* = 0.075), and medication treatment status (*p* = 0.168). However, the adult-diagnosed ADHD group exhibited a higher proportion of previous comorbid conditions (*p* = 0.012), particularly major depressive disorder. With respect to ADHD symptoms severity, the adult-diagnosed ADHD group had a lower number of hyperactive-impulsive childhood symptoms (p < 0.001) and inattentive childhood symptoms (p < 0.001) on the ADHD-RS compared with the childhood-diagnosed group. There were no differences between the two groups in the scores of inattention and hyperactivity-impulsivity (all *p* > 0.05) in adulthood. On the BRIEF-A, the adult-diagnosed group had lower scores of self-monitoring (*p* = 0.043), with no other significant differences in executive function domains. Furthermore, the adult-diagnosed group had more anxiety (*p* = 0.014) and depression symptoms (*p* < 0.001) than those in the childhood-diagnosed group. No significant group differences were found in resilience, childhood trauma exposure and parenting bonding (all *p* > 0.05).

**Table 1:** Demographic and clinical characteristics comparing adult-diagnosed and childhood-diagnosed ADHD groups.

Characteristics	Adult-diagnosed ADHD	Childhood-diagnosed ADHD	*P*-value
	(N = 35)	(N = 37)	
Age, years	25.63 ± 4.89	26.32 ± 4.69	0.54
Gender, n(%)			
Male	7 (20.0)	15 (40.5)	0.059
Female	28 (80.0)	22 (59.46)	
Educational years	16.41 ± 2.46	16.50 ± 1.73	0.858
Educational level			0.454
High school	1 (3.12)	2 (5.71)	
Bachelor’s degree	19 (59.38)	25 (71.43)	
Master’s degree	12 (37.50)	8 (22.86)	
Employment status			0.075
Student	16 (51.61)	10 (29.41)	
Employed	12 (38.71)	14 (41.18)	
Unemployed	3 (9.68)	10 (29.41)	
Previous comorbidities, n(%)			0.012*
Major depressive disorder	17 (48.6)	7 (18.9)	
Bipolar disorders	1 (2.9)	0 (0.0)	
Generalized anxiety disorder	2 (2.9)	1 (2.7)	
Obsessive-compulsive disorder	3 (2.9)	0 (0.0)	
Medication treatment, n(%)			0.168
Yes	8 (22.86)	14 (37.84)	
No	27 (77.14)	23 (62.16)	
ADHD-RS inattention	2.59 ± 1.92	7.00 ± 1.34	<.001*
ADHD-RS hyperactivity/impulsivity	1.45 ± 1.34	3.69 ± 2.19	<.001*
ASRS			
Inattention	27.34 ± 3.41	27.95 ± 4.61	0.532
Hyperactivity/impulsivity	16.34 ± 6.90	18.41 ± 7.89	0.243
BRIEF-A			
Inhibit	66.26 ± 11.60	68.24 ± 10.21	0.443
Emotional control	58.43 ± 9.82	58.68 ± 11.08	0.921
Initiate, M (Q_1_, Q_3_)	76.00 (72.00, 82.00)	76.00 (71.00, 79.00)	0.319
Working memory	75.86 ± 9.38	77.76 ± 8.91	0.381
Plan/organize	73.06 ± 11.11	72.51 ± 10.88	0.834
Organization of materials	66.37 ± 8.66	68.00 ± 8.66	0.428
Shift, M (Q_1_, Q_3_)	69.00 (62.50, 73.00)	69.00 (60.00, 74.00)	0.634
Self-monitor, M (Q_1_, Q_3_)	59.00 (48.00, 70.00)	64.00 (59.00, 72.00)	0.043*
Task monitor, M (Q_1_, Q_3_)	77.00 (68.50, 86.00)	77.00 (68.00, 81.00)	0.717
Behavioral regulation	64.69 ± 10.70	66.81 ± 10.12	0.389
Metacognition	77.03 ± 8.35	76.81 ± 8.96	0.915
Global executive composite	73.54 ± 8.95	74.35 ± 8.41	0.694
SAS	45.66 ± 8.69	41.00 ± 6.90	0.014*
SDS	50.97 ± 8.76	43.49 ± 8.29	<.001*
CD-RISC			
Resilience	77.94 ± 13.30	80.89 ± 13.49	0.365
CTQ			
Childhood trauma, M (Q_1_, Q_3_)	44.00 (33.50, 57.00)	41.00 (34.00, 50.00)	0.407
PBI			
Parental care	36.24 ± 9.59	37.97 ± 8.82	0.436
Parental overprotection	34.38 ± 5.18	34.00 ± 6.44	0.787

Data are presented as mean (SD) for normally distributed continuous variables, median (Q_1_, Q_3_) for non-normally distributed continuous variables, and frequency (n, %) for categorical variables. Q_1_ = 25th percentile; Q_3_ = 75th percentile. ∗ *p* < 0.05.

C, combined subtype; IA, inattention; ADHD-RS, ADHD rating scale; ASRS, adult ADHD self-report scale; BRIEF-A, Behavior Rating Inventory of Executive Function-Adult Form; SAS, Self-Rating Anxiety Scale; SDS, Self-Rating Depression Scale; CD-RISC, Connor-Davidson Resilience Scale, CTQ, Childhood Trauma Questionnaire; PBI, Parental Bonding Instrument.

### 3.2 Bivariate correlation analysis

**Figure 1:**
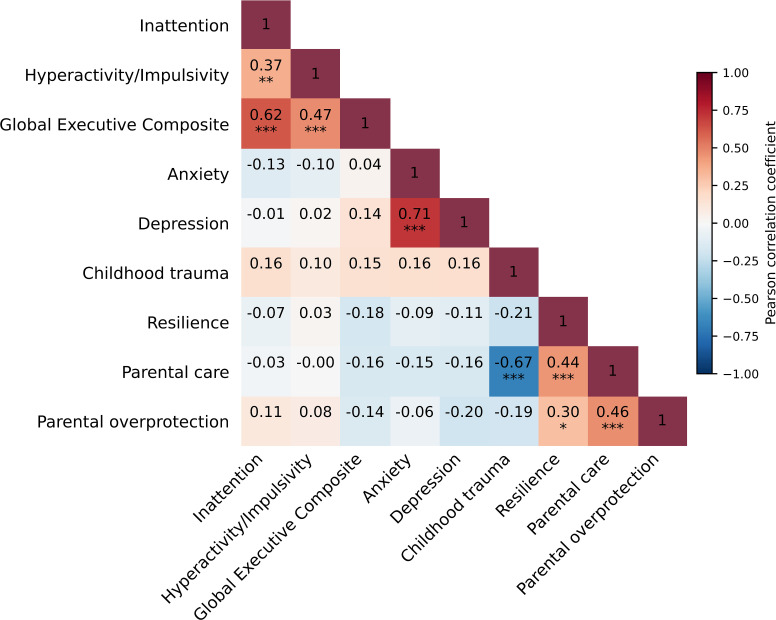
Bivariate correlations among the study variables. *p < 0.05, **p < 0.01, ***p < 0.001.

Correlations were examined for all study variables (see **Figure 1**). Executive function, as indexed by the GEC scores, was significantly positively correlated with inattention symptoms(*r* = 0.62, *p* < 0.001). In contrast, correlations between inattention and emotional or environmental factors were relatively weak (all *p* > 0.05). Hyperactivity-impulsivity symptoms were also positively correlated with executive function (*r* = 0.47, *p* < 0.001), whereas associations with emotional and environmental factors were minimal. Emotional and environmental factors showed a distinct pattern of associations. Anxiety symptoms and depression symptoms were strongly correlated (*r* = 0.71, *p* < 0.001). Parental care was inversely associated with childhood trauma (*r* = -0.67, *p* < 0.001) and positively associated with resilience (*r* = 0.44, *p* < 0.001). Parental overprotection was correlated with parental care (*r* = 0.46, *p* < 0.001) and resilience (*r* = 0.30, *p* < 0.05).

### 3.3 Hierarchical multiple regression analysis

To identify predictors of ADHD core symptoms, hierarchical regression analyses were performed (see **Table 2**). For inattention, none of demographic variables in Step 1, including age, gender, medication status, comorbidity, or age of diagnosis, significantly predicted inattentive symptoms. In Step 2, the addition of the GEC scores revealed that executive function emerged as a strong positive predictor of inattention (*β* = 0.64, *p* < 0.001), accounting for an additional 36.6% of the explained variance in inattention (Δ*R²* = 0.366, p < 0.001). Greater executive dysfunction was associated with more severe inattentive symptoms, an effect that remained robust after adjustment for anxiety and depressive symptoms in Step 3 (*β* = 0.64, *p* <.001), neither of which attained significance. In Step 4, environmental factors were added. While the overall increase in explained variance was not significant, parental overprotection was a significant predictor of inattention (*β* = 0.28, *p* = 0.016). In contrast, childhood trauma, resilience, and parental care were not significant. Executive function remained the strongest predictor in the final model, which explained 54.5% of the variance in inattention.

**Table 2:** Results of multivariate hierarchical regression analysis on ADHD core symptoms.

	Inattention				Hyperactive/impulsivity				
	*B*(SE)	*β*	*t* test (*df*)	*p*	*B*(SE)	*β*	*t* test (*df*)	*p*	
Step 1									
Age	0.20 (0.11)	0.24	1.88 (63)	0.064	0.07 (0.19)	0.05	0.39 (63)	0.699
Gender	-0.87 (1.13)	-0.10	-0.77 (63)	0.445	1.08 (2.00)	0.07	0.54 (63)	0.592	
Medication	-0.26 (1.12)	-0.03	-0.23 (63)	0.819	-0.69 (1.97)	-0.05	-0.35 (63)	0.727	
Comorbidity	-0.36 (1.13)	-0.04	-0.32 (63)	0.751	2.02 (2.00)	0.14	1.01 (63)	0.315	
Age of diagnosis	0.11 (1.07)	0.01	0.10 (63)	0.920	2.83 (1.89)	0.20	1.50 (63)	0.138	
Step 2									
Age	0.02 (0.09)	0.02	0.23 (62)	0.820	-0.16 (0.18)	-0.11	-0.88 (62)	0.382	
Gender	-0.61 (0.89)	-0.07	-0.68 (62)	0.496	1.41 (1.80)	0.09	0.79 (62)	0.435	
Medication	0.26 (0.88)	0.03	0.30 (62)	0.764	-0.03 (1.78)	0.00	-0.02 (62)	0.988	
Comorbidity	-1.53 (0.90)	-0.18	-1.70 (62)	0.095	0.53 (1.83)	0.04	0.29 (62)	0.775	
Age of diagnosis	-0.38 (0.84)	-0.05	-0.46 (62)	0.649	2.21 (1.70)	0.16	1.30 (62)	0.200	
GEC	0.31 (0.05)	0.64	6.38 (62)	<0.001*	0.39 (0.10)	0.47	4.01 (62)	<0.001*	
Step 3									
Age	0.03 (0.09)	0.04	0.37 (60)	0.710	-0.14 (0.18)	-0.09	-0.73 (60)	0.465	
Gender	-0.87 (0.89)	-0.10	-0.98 (60)	0.333	1.15 (1.84)	0.07	0.63 (60)	0.534	
Medication	0.69 (0.90)	0.08	0.76 (60)	0.449	0.46 (1.86)	0.03	0.25 (60)	0.805	
Comorbidity	-1.46 (0.90)	-0.17	-1.62 (60)	0.111	0.69 (1.86)	0.05	0.37 (60)	0.712	
Age of diagnosis	-0.90 (0.94)	-0.11	-0.95 (60)	0.344	1.86 (1.94)	0.13	0.96 (60)	0.342	
GEC	0.31 (0.05)	0.64	6.28 (60)	<0.001*	0.38 (0.10)	0.46	3.81 (60)	<0.001*	
Anxiety	-0.10 (0.07)	-0.20	-1.39 (60)	0.170	-0.14 (0.15)	-0.16	-0.97 (60)	0.334	
Depression	0.01 (0.06)	0.02	0.12 (60)	0.908	0.05 (0.13)	0.07	0.40 (60)	0.689	
Step 4									
Age	0.11 (0.09)	0.12	1.12 (56)	0.268	-0.04 (0.21)	-0.03	-0.19 (56)	0.854	
Gender	-1.15 (0.89)	-0.13	-1.29 (56)	0.203	1.42 (1.94)	0.09	0.73 (56)	0.469	
Medication	0.59 (0.92)	0.07	0.65 (56)	0.521	0.15 (2.00)	0.01	0.08 (56)	0.940	
Comorbidity	-1.30 (0.90)	-0.15	-1.44 (56)	0.156	1.10 (1.96)	0.08	0.56 (56)	0.577	
Age of diagnosis	-0.52 (0.91)	-0.06	-0.57 (56)	0.569	2.30 (1.99)	0.16	1.15 (56)	0.253	
GEC	0.30 (0.05)	0.62	6.27 (56)	<0.001*	0.38 (0.10)	0.46	3.65 (56)	<0.001*	
Anxiety	-0.14 (0.07)	-0.27	-1.95 (56)	0.057	-0.17 (0.15)	-0.20	-1.13 (56)	0.264	
Depression	0.05 (0.06)	0.12	0.86 (56)	0.395	0.10 (0.14)	0.13	0.69 (56)	0.492	
Childhood trauma	0.04 (0.04)	0.14	1.03 (56)	0.309	0.06 (0.08)	0.12	0.70 (56)	0.486	
Resilience	-0.02 (0.03)	-0.08	-0.69 (56)	0.492	0.05 (0.07)	0.09	0.66 (56)	0.513	
Parental care	0.04 (0.07)	0.09	0.57 (56)	0.569	0.01 (0.15)	0.02	0.09 (56)	0.929	
Parental overprotection	0.20 (0.08)	0.28	2.49 (56)	0.016*	0.16 (0.17)	0.13	0.94 (56)	0.351	

*R²* values correspond to the total variance explained by the model at each step. Δ*R²* values correspond to variance explained by each step. ∗ *p* < 0.05.

*B* (SE), unstandardized coefficient and its standard error; *β*, standardized coefficient; GEC, global executive composite.

Model summary (Inattention): Step 1, *R²* = 0.077; Step 2, *R²* = 0.442, ΔR² = 0.366, *F*(1,62) = 40.68, *p* <.001; Step 3,*R²* = 0.470, Δ*R²* = 0.028, *F*(2,60) = 1.57, *p* = 0.217; Step 4, *R²* = 0.545, Δ*R²* = 0.075, *F*(4,56) = 2.31, *p* = 0.069.

Model summary (Hyperactive/impulsivity): Step 1, *R²* = 0.044; Step 2, *R²* = 0.241, ΔR² = 0.197, *F*(1,62) = 16.11, *p* <.001; Step 3, *R²* = 0.254, Δ*R²* = 0.013, *F*(2,60) = 0.53, *p* = 0.591; Step 4, *R²* = 0.286, Δ*R²* = 0.032, *F*(4,56) = 0.62, *p* = 0.65.

Regarding hyperactivity-impulsivity, Step 1 showed that demographic variables did not significantly predict symptom severity. In Step 2, executive function was a significant predictor (*β* = 0.47, *p* < 0.001), accounting for an additional 19.7% of the variance in hyperactivity-impulsivity (Δ*R²* = 0.197, *p* < 0.001). This association remained significant in Step 3 after adjustment for anxiety and depressive symptoms, which did not contribute significantly to the model. In Step 4, the inclusion of environmental factors did not significantly improve model fit, and none of these factors reached significance. In the final model, executive function remained the only consistent predictor of hyperactivity-impulsivity, which explained 28.6% of the variance in symptom severity.

Taken together, executive function explained a substantial proportion of variance in both inattention and hyperactivity-impulsivity symptoms, whereas environmental factors contributed limited additional explanatory power after executive function was taken into account.

### 3.4 Moderation effects

Moderation analyses examined whether age of diagnosis moderated the associations between executive function, environmental factors, and ADHD core symptoms. For inattention, none of the interaction terms were significant. The interaction between executive function and age of diagnosis showed a marginal significance (*β* = 0.25, *p* = 0.077), but did not significantly improve explained variance (Δ*R*² = 0.025). Interactions involving childhood trauma, resilience, parental care, and parental overprotection were also non-significant, accounting for minimal additional variance (Δ*R*² ≤ 0.013). For hyperactivity-impulsivity, no significant interaction effects were observed (all *p* > 0.05). Overall, there was no difference in the associations of executive function and environmental factors with ADHD core symptoms between adult-diagnosed and childhood-diagnosed ADHD groups (**Table 3**).

**Table 3:** Moderated effects: different age of diagnosis as a moderator in the associations between executive function, environmental factors, and ADHD core symptoms.

Interaction	*B*(SE)	*β*	*t* test(*df*)	*p*	95% CI	*R²*	*ΔR²*
Inattention							
GEC × age of diagnosis	0.17 (0.10)	0.25	1.80 (55)	0.077	-0.04, 0.37	0.570	0.025
Childhood trauma × age of diagnosis	0.07 (0.06)	0.17	1.25 (55)	0.217	-0.08, 0.19	0.558	0.013
Resilience × age of diagnosis	-0.04 (0.06)	-0.09	-0.64 (55)	0.526	-0.18, 0.07	0.548	0.003
Parental care × age of diagnosis	-0.08 (0.09)	-0.13	-0.96 (55)	0.341	-0.26, 0.12	0.553	0.008
Parental overprotection × age of diagnosis	0.03 (0.14)	0.03	0.22 (55)	0.823	-0.27, 0.33	0.546	0.000
Hyperactive/impulsivity							
GEC × age of diagnosis	0.08 (0.21)	0.07	0.38 (55)	0.707	-0.32, 0.55	0.288	0.002
Childhood trauma × age of diagnosis	0.19 (0.12)	0.27	1.59 (55)	0.117	-0.12, 0.46	0.317	0.031
Resilience × age of diagnosis	0.06 (0.14)	0.08	0.43 (55)	0.667	-0.24, 0.31	0.288	0.002
Parental care × age of diagnosis	0.12 (0.19)	0.10	0.60 (55)	0.548	-0.27, 0.47	0.290	0.005
Parental overprotection × age of diagnosis	-0.07 (0.30)	-0.05	-0.24 (55)	0.812	-0.67, 0.72	0.286	0.001

Each interaction term was entered after all main effects, including executive function, emotional symptoms, and environmental factors, were controlled.

Different age of diagnosis was coded as 0 = adult-diagnosed ADHD and 1 = childhood-diagnosed ADHD.

*R²* values correspond to the total variance explained by the model at each step. *ΔR²* values correspond to the additional variance explained by the interaction term.

*B* (SE) = unstandardized coefficient and its standard error; *β*: standardized coefficient; GEC, global executive composite.

## 4 Discussion

In this study, we characterized the demographic and clinical features of adults with adult-diagnosed ADHD, comparing them to childhood-diagnosed ADHD. Our results showed that the adult-diagnosed ADHD group had less severe symptoms during childhood compared with the childhood-diagnosed group. The two groups showed comparable symptom severity in adulthood. Notably, adult-diagnosed ADHD was characterized by distinct psychosocial features, including superior self-monitoring and higher levels of anxiety and depressive symptoms. Additionally, this study investigated whether executive function and environmental factors contribute differentially to ADHD core symptoms across different age of diagnosis. The findings revealed a largely convergent pattern of associations rather than distinct predictor profiles for each group. Across models, symptom severity correlated more strongly with executive function than with emotional or environmental variables. Moderation analyses further indicated that these associations did not differ significantly according to the age of diagnosis.

Although gender differences were not statistically significant, the trend toward a higher proportion of females in the adult-diagnosed ADHD group aligns with previous findings suggesting that ADHD in females is frequently identified later in life (28–30). This diagnostic delay may be attributed to a sex-stratified “masking effect”, wherein females often present with predominantly inattentive and internalizing symptoms, such as anxiety or depression, rather than overt hyperactivity (31–35). Consequently, these clinical presentations render ADHD less recognizable during childhood. Furthermore, it is plausible that superior cognitive compensation and social adaptation strategies enable these individuals to mask difficulties until the escalating academic or occupational demands of adulthood exceed their compensatory capacity, thereby triggering evident functional impairment (36, 37).

Executive function emerged as the most robust and consistent correlate with inattentive and hyperactive-impulsive symptoms, and this association remained robust after controlling for demographic characteristics, emotional symptoms, and environmental factors. This finding is consistent with meta-analytic evidence identifying executive deficits as the core impairments of adult ADHD and extends prior work by demonstrating that this association is comparable across adult-diagnosed and childhood-diagnosed presentations, supporting the significance of executive dysfunction in ADHD (38, 39). With the ongoing debate concerning the validity and nature of adult-diagnosed ADHD, the present results indicate that executive function represents a shared cognitive-functional pathway underlying symptom expression in adulthood rather than a mechanism specific to childhood-onset cases. Consequently, the results suggest that assessment and intervention strategies targeting executive function may be equally relevant for adults with adult-diagnosed and childhood-diagnosed ADHD.

Our results indicated that adult-diagnosed ADHD exhibited a higher burden of psychiatric comorbidities, particularly internalizing disorders. This finding aligns with existing literature suggesting that individuals diagnosed with ADHD in adulthood frequently seek medical attention due to emotional or anxiety symptoms (40, 41). Such symptoms often mask the underlying attentional difficulties during clinical presentations, thereby leading to delayed diagnosis and more severe functional impairments across academic and social domains (10). Two competing causal pathways may explain this association. First, the cumulative frustration arising from longstanding unidentified executive function deficits may exacerbate the risk of developing secondary internalizing disorders (42). Alternatively, primary mood or anxiety disorders may themselves manifest as attentional and regulatory difficulties that resemble ADHD, leading individuals to receive a diagnosis in adulthood in the absence of an independent neurodevelopmental condition (43). The potential for misdiagnosis warrants explicit consideration given the substantial overlap between ADHD and internalizing disorders. Major depressive disorder impairs sustained attention and working memory, while generalized anxiety disorder generates attentional dyscontrol that closely parallels inattentive ADHD (10, 44). In this research, the use of the MINI facilitated screening for current active psychiatric disorders, while the CAADID required that symptoms demonstrate cross-situational persistence and remain distinguishable from episodic mood disturbances. Nevertheless, these procedural safeguards focus on the current psychiatric status rather than the longitudinal trajectory of symptoms; consequently, they cannot fully resolve the ambiguity introduced by a history of recurrent affective episodes. Although emotional symptoms did not independently predict the severity of ADHD after accounting for executive function in the present analysis, the high prevalence of these comorbidities remains a critical factor in clinical differentiation (45). It is essential for clinicians to distinguish between the stable cognitive impairments of ADHD and the more transient or fluctuating affective states common in mood disorders. Recognizing these overlaps is vital for ensuring diagnostic accuracy and appropriate treatment implications for adult populations (46).

Although parental overprotection showed a modest association with inattentive symptoms, its effect was small and secondary to executive function. It has been proposed that adult-diagnosed ADHD might be underrecognized or less impairing during childhood due to protective factors, such as high cognitive strengths or strong environmental support (6, 47). In this regard, early environmental scaffolding, such as structured parental attention or supportive school environments, may have compensated for executive function deficits during childhood. As these individuals transition into adulthood and encounter reduced external scaffolding alongside heightened demands for autonomy, this compensatory mechanism may fail, thereby leading to an adult-diagnosed clinical presentation (6, 47). Additionally, cognitive and memory biases warrant careful consideration when interpreting the observed group differences. Adult-diagnosed individuals may over-report current and retrospective symptom severity due to negative cognitive biases (43); conversely, they may also underreport childhood difficulties owing to the absence of a prior diagnostic framework (48). While childhood trauma is generally associated with an increased risk of ADHD (12), our moderation analyses did not reveal significant interactions between the age of diagnosis and environmental factors, including childhood trauma, resilience, and parental bonding, for either inattentive or hyperactive-impulsive symptoms. This may suggest the possibility that both groups share a common neurodevelopmental underpinning (11, 49). However, these findings should be interpreted with caution. Due to the modest sample size and the cross-sectional nature of this study, we cannot definitively exclude the possibility of environmental protective effects or establish causal relationships (4). The lack of a significant impact from childhood trauma in our models might also be constrained by these power limitations. The potential for environmental scaffolding to mask symptoms remains a crucial consideration in clinical practice and future longitudinal research.

There are several limitations in this study. Firstly, as a cross-sectional study, this design precludes causal or directional inferences, and it relied on retrospective reports of ADHD symptom-onset and psychiatric history to exclude alternative explanations for adult-diagnosed symptoms. Secondly, the modest sample size may limit the generalizability of the findings. Thirdly, adult-specific measures were self-reported, which may introduce recall or reporter bias; in particular, executive function was assessed using self-report measures, which may capture perceived difficulties rather than objective cognitive capacity. Finally, although we controlled for current stimulant use, the lack of data on dosage, duration, and other medications limited our ability to fully assess medication effects on symptoms. Therefore, future longitudinal research is needed to better delineate causal pathways in adults with ADHD with multi-informant ratings and objective cognitive assessment.

In conclusion, our study addresses a critical gap of adult ADHD literature by clarifying whether executive function and environmental factors differentially relate to symptom severity across adult diagnosed and childhood diagnosed ADHD. The findings indicate a largely convergent pattern across different age of diagnosis, with executive dysfunction emerging as a shared and robust correlate of symptom severity independent of onset history, alongside a limited and nonspecific explanatory role of environmental factors. Consequently, environmental factors and executive function do not constitute a primary, diagnosis-specific mechanism underlying adult-diagnosed ADHD.

## Data Availability

The original contributions presented in the study are included in the article/supplementary material. Further inquiries can be directed to the corresponding authors.
